# ‘It's Hard to Look Past the Accessibility’: Service Provider Perspectives on Implementing Telehealth for Alcohol and Other Drug Counselling as a Health Service Innovation

**DOI:** 10.1111/dar.70000

**Published:** 2025-06-19

**Authors:** Ashlea Bartram, Md Abdul Ahad, April Long, Dan I. Lubman, Ele Morrison, Jill Rundle, Nicole Lee, Scott Wilson, Jacqueline Bowden

**Affiliations:** ^1^ National Centre for Education and Training on Addiction, Flinders Health and Medical Research Institute Flinders University Adelaide Australia; ^2^ College of Medicine and Public Health, Flinders Health and Medical Research Institute Flinders University Adelaide Australia; ^3^ Smart Recovery Australia Sydney Australia; ^4^ Monash Addiction Research Centre and Eastern Health Clinical School, Faculty of Medicine, Nursing and Health Sciences Monash University Melbourne Australia; ^5^ Turning Point, Eastern Health Melbourne Australia; ^6^ Australian Injecting and Illicit Drug Users League Sydney Australia; ^7^ Western Australian Network of Alcohol and Other Drug Agencies Perth Australia; ^8^ Hello Sunday Morning Sydney Australia; ^9^ National Drug Research Institute Curtin University Perth Australia; ^10^ Aboriginal Drug and Alcohol Council Adelaide Australia

**Keywords:** Australia, counselling, substance‐related disorders, telemedicine

## Abstract

**Introduction:**

Offering telehealth services is an opportunity to reduce barriers to accessing and attending face‐to‐face alcohol and other drug (AOD) treatment. However, little is known about how telehealth options have been implemented by AOD services.

**Methods:**

Key informant interviews were conducted with 20 senior staff members from government and non‐government AOD treatment services in Australia with current or previous experience in delivering AOD counselling face‐to‐face and via phone and/or video, and thematically analysed.

**Results:**

Interviewees described implementing telehealth flexibly alongside face‐to‐face counselling, with choice of mode shaped by client needs and preferences. Telehealth was valued for increasing the service's ability to reach under‐served clients and for allowing clients to access services from a place of psychological safety. However, service providers were less certain about the comparative safety and effectiveness of delivering counselling through telehealth, especially when delivered over the phone. Telehealth was viewed as relatively compatible with current processes for face‐to‐face delivery, including processes for staff skill development.

**Discussion and Conclusions:**

Telehealth has been implemented alongside face‐to‐face counselling to increase access and meet client preferences. Further evidence regarding risk assessment and client outcomes via phone and video is needed to support broader diffusion of this innovation.

## 1 Introduction

Over 131 500 Australians accessed treatment for alcohol and other drug (AOD) use in 2022–23 from publicly funded AOD treatment providers [1], with many more seeking assistance privately from general practitioners, addiction or mental health professionals. Despite this, the unmet need for AOD treatment services in Australia is substantial, with estimates that only 30%–48% of the people who would benefit from AOD treatment are able to access it [2]. This situation is not unique to Australia: a review of studies internationally found that the median treatment gap for alcohol dependence was 78% [3]. Key barriers to accessing treatment include shame and stigma, low perceived treatment need, and structural barriers such as financial, geographical and wait‐time constraints; structural barriers in particular were predominant in regional settings [4]. There is considerable interest in service innovations that might increase access to AOD support, including through telehealth [5]—which is the focus of this paper.

Telehealth can be defined as a health service offering delivered to an individual client in real time, where the clinician is located remotely from the client [5]. It commonly includes services delivered via phone or video call, but can also include approaches such as online synchronous text‐based chat support [6, 7]. Systematic reviews have found telehealth to be an effective mode of delivery across a range of health services, including primary care triage [8], musculoskeletal management [9], and mental health treatment [10, 11]. A synthesis of systematic reviews concluded that telehealth appears to be similarly effective to face‐to‐face services, particularly for services based on verbal assessment and when clients and health service providers have an existing relationship [12]. Evidence specific to AOD services is more limited. A systematic review of telehealth for a range of mental health conditions including substance dependence [13] identified two randomised controlled trials in which adult outpatients receiving opioid dependence treatment in the United States received counselling either in person or via videoconferencing, with similar outcomes in both conditions [14, 15]. Standalone telephone counselling services have also been effective in reducing substance use problem severity [16], particularly among clients who attend at least two sessions [17].

Outside of the context of controlled trials and stand‐alone services, the uptake and implementation of health service innovations such as telehealth can be inconsistent: services may adopt an innovation to varying extents, implementing it using varying (and not always clearly specified) protocols both across services and from clinician to clinician within services [18, 19]. Diffusion of innovation theory proposes that innovations are likely to be adopted when they provide a comparative advantage to the status quo, are compatible or consistent with current processes, contexts and values, easy to understand and use, and easy to trial or apply flexibly [20]. Decision‐makers in health care may consider both the workability of an intervention, in terms of its comparative advantage, and its capacity for integration within existing (or new) health services [18]. For health technologies, a comparative advantage can be in terms of its clinical‐ or cost‐effectiveness, safety, and accessibility and acceptability for clients [21]. As well as the nature of the innovation and its advantage(s), the implementation of an innovation is shaped by the individuals involved, the activities and strategies used to implement it, and the organisational and broader environmental contexts in which the innovation is implemented [22].

The advent of the COVID‐19 pandemic and its associated restrictions on in‐person interactions—a substantial change in environmental context—led to a rapid uptake of telehealth across health systems, including within the AOD sector [23, 24, 25]. Services pivoted to provide clients access to AOD treatment options via telehealth [23, 24], which were found to be accessible, feasible, and effective [24, 26, 27, 28]. However, clinicians reported finding it more difficult to establish therapeutic relationships and make clinical assessments based on few or no visual cues [25, 27, 29]. In addition, telehealth may exacerbate a digital divide, with many AOD clients coming from sociodemographic groups that are digitally excluded, such as the unemployed, people who are homeless, and people from regional and remote areas with limited digital infrastructure [25, 30].

Since the easing of pandemic restrictions, face‐to‐face services have resumed, but many AOD organisations have continued to offer telehealth appointments alongside these services [23, 25]. Little is known about the implementation of telehealth AOD services in a post‐pandemic context in which many clients and clinicians have become more familiar with digitally mediated interactions. In this study, we investigated the experiences of Australian AOD specialist treatment services in implementing telehealth counselling services alongside face‐to‐face services. We focused on counselling as the most common AOD treatment type in Australia [1], and one for which telehealth has demonstrated effectiveness comparable with face‐to‐face delivery in related fields such as smoking cessation [31] and mental health [10, 11]. Drawing on senior staff members as key informants [32], we addressed the following research question: What are the perspectives of senior staff from Australian AOD services that have experience offering both telehealth and face‐to‐face counselling on the implementation of telehealth for AOD counselling?

## 2 Methods

### 2.1 Study Design

We employed a key informant interview study design under a realist/essentialist epistemological framework, viewing the aim of the research as understanding and reporting the experiences, meaning, and reality of participants with in‐depth knowledge about a topic [32, 33]. Key informants were senior staff members employed by Australian AOD services that offer face‐to‐face counselling and had either currently or previously offered counselling via telehealth.

### 2.2 Procedures

Ethics approval was obtained from the Human Research Ethics Low Risk Panel at Flinders University (No. 6516). Participants were purposefully recruited via AOD peak representative bodies from each Australian state and territory (‘Peaks’). We asked each Peak to share information about their study with member AOD treatment organisations that had experience in delivering counselling via telehealth; some directly approached member organisations that they knew had relevant experience (information rich cases) while others shared information broadly via their electronic newsletters. Peaks primarily represent non‐government treatment services. Thus, a limitation of this approach meant that participants predominantly represented non‐government services. One government service provider was included after a representative learned about the study from a Peaks newsletter and contacted the research team.

Interviews were conducted by AB via Microsoft Teams between November 2023 and May 2024. All participants were given study information and provided informed consent prior to the interview. Interviews were semi‐structured and ran for approximately 1 h; interview topics included the organisation's processes of using telehealth; benefits, drawbacks, and challenges; the participant groups for whom telehealth works best; key learnings or advice for others; and areas where there is a need for more research. No reimbursement was provided as participants took part in interviews as part of their paid employment. No participants requested their interview to cease and/or their data to be withdrawn.

### 2.3 Analysis

Interviews were recorded and automatically transcribed in Microsoft Teams, with transcripts de‐identified and reviewed for accuracy by a research assistant then imported into NVivo (release 1.3) for analysis [34]. We took a reflexive thematic approach to analysis, following the guidelines suggested by Braun and Clarke [33]. AB first read and re‐read transcripts to gain familiarity with the data, then systematically coded features of the data relating to the research question across the entire data set; coding was semantic and codes were developed inductively through the analysis process. To develop candidate themes, AB used NVivo to create a visual map of codes, grouping similar codes together and denoting connections between groups of codes. AB then used this map to guide the development of candidate themes and subthemes. All authors reviewed the thematic structure and related data extracts. The authors comprised people with diverse backgrounds in AOD research, service design and administration, clinical practice, policy development, advocacy, and lived and living experience of AOD and service use; many of us also have personal experience of using telehealth services in a range of healthcare contexts. Our varied backgrounds and perspectives have informed our interpretation of the data, orienting us towards literature on healthcare innovation and health technology assessment; we drew on this literature to inform theme development and naming, including concepts relating to the comparative advantage of healthcare innovations (accessibility, safety, and effectiveness) and its compatibility with organisational processes [18, 20, 21, 22]. We resolved any disagreements through discussion, focusing on ensuring that our final themes and interpretations were grounded in the data. We have presented quotes from participants to illustrate each key theme. To protect the anonymity of respondents, each quote is attributed to a numeric participant identifier as well as noting whether the service the participant represented was from a metropolitan location, a regional location or both a metropolitan and regional location (‘both’).

## 3 Results

Interviewees were 20 staff members (11 female, 9 male) representing 17 service providers across Australia. Interviewees held a mix of managerial (*N* = 7), counselling (*N* = 9) or combined managerial and counselling (*N* = 4) positions. Most interviewees were based in South Australia (*N* = 7), followed by Victoria (*N* = 5), the Northern Territory (*N* = 3), New South Wales (*N* = 2), and Western Australia (*N* = 2). One service provider was a government organisation; all others were non‐government organisations. Service providers operated in metropolitan areas (*N* = 4), regional areas (*N* = 3), or both metropolitan and regional (*N* = 10). Three service providers were Aboriginal community‐controlled organisations.

Interviewees reported that their services utilised telehealth to varying degrees, with estimates ranging from ‘A tiny fraction … less than 1%’ (Interview 1, metropolitan), to ‘About 80%’ (Interview 9, both metropolitan and regional [henceforth ‘both’]); phone was more commonly utilised than video. Most described a hybrid implementation of telehealth, with clients potentially switching between phone, video, and face‐to‐face across the course of their engagement with the service. Major themes related to the accessibility of the service, perceptions of effectiveness and safety, and approaches to integrating telehealth alongside face‐to‐face services.

### 3.1 Theme 1: Telehealth Improves the Accessibility of Services

Interviewees overwhelmingly identified the capacity for telehealth to increase access to AOD counselling for clients as its primary value. This included increasing access for people who lived long distances from services, had limited access to transport, whose caring or employment responsibilities limited their availability during business hours, and those facing physical or mental health challenges that made it difficult to travel. For example:

‘It gives people access … it just makes it a whole lot easier from a client's perspective. For a lot of the clients [we] might only be 5 or 6 kilometres away, but that 5 or 6 kilometres and $2.00 for a bus ticket can actually be a bit of a barrier for some people. Several of the clients on my list have medical issues as well, which makes mobility difficult’. (Interview 14, regional).

Offering telehealth also supported service providers from regional areas to provide access to counselling in settings where they were constrained in their capacity to provide face‐to‐face services due to their large, geographically dispersed catchments of clients. Some services noted telehealth had reduced their staff travel times, allowing staff to spend more time with clients:

‘There's value in your clinicians not driving around the place. There's value in not needing as big a pool of work, cars, and mileage and them not driving an hour to an appointment and an hour back’. (Interview 3, both).

Interviewees also noted that telehealth could supplement their service's face‐to‐face services in locations they only had the capacity to attend in‐person infrequently:

‘We've got clinicians that have got particular regions that they outreach to and it might be fortnightly visits to some of the areas. So telehealth is really important in just being able to provide those additional sessions in between the physical outreach sessions’. (Interview 6, regional).

Services were sometimes able to provide access to counselling more rapidly by using telehealth, bypassing waiting lists for face‐to‐face services in high demand areas and providing a more equitable service:

‘Being able to share referrals across my team, so that we can balance how long people are waiting to access treatment and it's more equal and fair. And that just because someone's in a regional area, for example, they don't have to wait triple the amount of time that someone in a metro area has to wait’. (Interview 10, both).

Telehealth could also support continuity of access to counselling for clients as their life circumstances changed; for example, interviewees described clients who had relocated, entered employment, or had children and used telehealth to maintain access to the counsellor with whom they had established a relationship.

Broadly, telehealth was seen as supporting flexible engagement with clients, led by clients’ preferences and needs; many clients reportedly used a mix of telehealth and face‐to‐face services. However, interviewees were also clear that telehealth was not for everyone, with some clients reportedly preferring, or seeming to engage better, face‐to‐face. Interviewees were reluctant to make generalisations based on demographics or substance use, noting that individual differences were considerable: ‘I don't like putting anyone into categories’ (Interview 2, both). Interviewees also expressed conflicting perspectives. Several interviewees suggested that clients with multiple complexities were best seen face‐to‐face so that their wellbeing could be more closely monitored. However, one interviewee who worked exclusively with clients with ‘more complex clinical pictures’ offered a counter perspective, noting that telehealth supported his clients to access counselling:

‘People who had comorbid mental health problems, and a lot of them had social phobia, social anxiety … memory problems … the clients we're seeing are more likely to have alcohol related brain injury or traumatic acquired brain injury as well. So getting here sometimes was an issue for them, even just knowing how to get here. So the characteristics of our clients really lends itself quite well to telephone counselling’. (Interview 12, metropolitan).

Interviewees did note that not all clients had access to resources for telehealth to be effective for them, such as phone or internet data, a reliable connection, comfort with technology, and a private space from which to speak with their counsellor:

‘[The people who loved telehealth]: These are people who have money, have resources, have access to tech, turn up on time … If you don't have that stuff, if you run out of credit, then it's no good for you. Or if you've got nowhere private to sit or, you know, you're not kind of warm and comfortable where you are’. (Interview 7, both).

Telehealth was thus seen in the most part as an adjunct to, not a replacement for, face‐to‐face services: increasing access for some clients but reportedly difficult to access for others.

### 3.2 Theme 2: Accessing Counselling via Telehealth is More Effective Than Accessing Nothing

Only four interviewees noted that their service had formally evaluated the use of telehealth, seeking feedback from clients and/or examining their organisation's data around telehealth utilisation, but all offered their impressions on the effectiveness of telehealth. Many interviewees believed that telehealth improved attendance, with one interviewee providing statistics to demonstrate:

‘Our attendance rates shifted quite dramatically: Face to face … about 49%, whereas over the phone we had … 63%, … video was 59%’. (Interview 17, both).

Interviewees were more hesitant regarding their perceptions of the effectiveness of conducting counselling over phone or video. Their hesitance was largely shaped by two factors: challenges in establishing rapport via telehealth, and perceptions that clients did not take telehealth appointments as seriously as face‐to‐face appointments: ‘just having a chat … just a catch up’ (Interview 17, both). With respect to rapport, the lack of visual and other sensory cues was felt to make engaging with clients difficult:

‘You don't have a direct line of vision, the ability to read body language. Something that I've been concerned about is whether clients are getting the most out of this, if the rapport is there or if it just feels like they just talked to some stranger over the phone’. (Interview 15, metropolitan).

Interviewees noted that these concerns were not as pronounced with video platforms, but that clients' preferences for phone calls meant that video was underutilised.

Interviewees noted that counsellors could become very good at engaging clients and building rapport over telehealth—although it could require a different approach to face‐to‐face:

‘I have noticed that it's quite a different way of counselling. That's what some training probably needs to be about, building rapport on telehealth or picking up on cues on telehealth. From what I've experienced, you have to be a lot more directive or assertive about checking in with the client more often, how they're tracking and how they're going, just because you just can't sense’. (Interview 6, regional).

Some interviewees also noted that telehealth provided some therapeutic advantages over face‐to‐face counselling. For example, one interviewee explained how he leveraged the fact that clients were not constrained to be in a counselling office:

‘I can plan ahead. “So listen, why don't you go down to your local park or down the beach or whatever? We could do a mindfulness exercise in real life.” So that gives people a real‐life example of how to do a bit of that sort of thing, which just wouldn't happen in a counselling room, which is a very artificial environment’. (Interview 9, both).

Given the increased reach and perceived improvements in attendance afforded by telehealth, nearly all interviewees were of the view that undertaking AOD counselling via telehealth was at the very least, better than accessing no counselling: ‘I mean, getting something is more effective than nothing.’ (Interview 9, both). However, many interviewees wanted to see more evidence on outcomes from undertaking telehealth counselling—noting that such evidence had been difficult for them to generate:

‘We don't have enough [outcome data] … we're a fair way off that, we're just trying to get basic outcomes stuff at the moment, let along breaking it down [by mode of delivery]’. (Interview 7, both).

### 3.3 Theme 3: Telehealth May Increase Clients' Sense of Safety but May Pose Challenges for Privacy, Clinical Assessment and Monitoring

Interviewees expressed conflicting perspectives on the safety of conducting AOD counselling via telehealth, acknowledging that clients may find telehealth a psychologically safe way to access counselling but raising concerns with the capacity of counsellors to manage privacy, conduct clinical assessments, and monitor risk.

The option for clients to undertake an appointment from a place of their choosing, often their own home, was seen as increasing clients’ comfort and willingness to engage in counselling. Interviewees noted the stigma and shame associated with seeking AOD counselling, which could be heightened by physically attending a clinic, where clients might be seen by others:

‘There is a lot of shame and stigma … having telehealth as an option and not having to … walk into an organisation that specifically treats clients [from a stigmatised community], it really does open up a door for people’. (Interview 15, metropolitan).

Interviewees reported that they predominantly provided telehealth services via phone, which they reasoned provided the greatest sense of psychological safety due to its absence of visual cues. Many interviewees believed that clients disliked using video platforms because they did not feel confident with how they appeared: ‘It's really confronting, I think, to see yourself on video, particularly if you already struggle with guilt and shame and low self‐esteem, which we kind of know goes with addiction’ (Interview 4, regional). Interviewees also noted that phone counselling could provide some perceived distance from the counsellor, and thus be an easier entry point for people who were less comfortable with the concept of talk‐based therapies:

‘Particularly clients from different backgrounds who don't do counselling in their culture … what I've found is being on the phone has put in a bit of a, I suppose a physical barrier to the extent that they don't feel so on the spot, or uncomfortable to talk’. (Interview 9, both).

Interviewees noted however that telehealth did not always live up to the potential for improved psychological safety for clients, as clients were not always in a private location for their sessions. For example:

‘There have been cases where I've called up and they've been in a car with their mates or something like that. I said, “Listen, this is not appropriate.” … So I will send a text and say please make sure you're in a space that's private, in the text message with the reminder’. (Interview 9, both).

Noting limitations with phone calls, some services encouraged clients to move to video platforms as their confidence improved:

‘I've had a few clients that don't feel comfortable with video initially, don't want to come in face‐to‐face and so they just request telephone. But then a couple of sessions in I'll check in and I'm like “Would you like to try video next time?”. And then, you know, seeing their confidence grow and just, you know, communication skills and all those kinds of skills, I think that was [an] unexpected [benefit of telehealth] for me’. (Interview 6, regional).

Although telehealth was seen as supporting clients' feelings of safety, interviewees reported that the absence of visual and other sensory cues made it more difficult for the counsellor to monitor their clients' safety and assess risk. For this reason, several interviewees noted that they preferred clients to attend an initial appointment face‐to‐face:

‘We are trying to encourage clients to come face‐to‐face to a first appointment, even if they prefer telehealth. If it's possible geographically and they can come in, we're preferring if they can … so we can meet them and get a good sense of their presentation’. (Interview 10, both).

Despite concerns about monitoring safety over telehealth, many interviewees also noted that clients were more likely to answer a phone call than attend face‐to‐face counselling if they were in crisis, providing an opportunity to provide support at a critical moment:

‘Some clients who are in crisis, they would not show up if there wasn't another option. So [telehealth] gives me access to them when they are not doing so well … For example, a couple of weeks ago a client in crisis called five minutes before and said “Can we do an over the phone instead?” And she was hyperventilating, very heightened throughout the session and I was able to run her through breathing techniques … I wouldn't have seen her otherwise’. (Interview 17, both).

This opportunity to reach clients in crisis meant that considerations of how to manage emergency situations over telehealth were important. Broadly, interviewees reported that their processes for managing emergencies mirrored those used for face‐to‐face encounters (‘It's the same sort of procedure’ (Interview 12, metropolitan), although they might be more likely to contact emergency services:

‘We do sometimes have to err on the side of caution of contacting emergency services or welfare checks of that kind of thing because we're a little bit limited in our ability to fully assess somebody's risk, whether that's substance use or self‐harm or risk to others’. (Interview 10, both).

### 3.4 Theme 4: Telehealth Can be Implemented in Parallel to In‐Person Services

When asked about the processes for conducting counselling via telehealth in their service, interviewees consistently noted that these mirrored processes for face‐to‐face services. Interviewees generally described only minor adjustments to processes, such as introducing verbal or electronic consent processes, or using video platforms, e‐mail, or text to share resources that they might have provided physically in face‐to‐face sessions. Some interviewees reported providing additional guidance to clients to explain how their counselling session would work via telehealth, including instructions for accessing the platform and what to do if they encountered technical difficulties. For example:

‘We set up expectations in the first session in telehealth similar as we would face‐to‐face. So, we talk about attendance and non‐attendance and what are the implications of non‐attendance the same as we would face‐to‐face. But I guess with telehealth, there's some differences that you'd talk about like the space that the client is in when they accept the call or the Zoom, like they need to be in a private confidential space. What happens if the call cuts off? How do we manage risk? What if we're worried about them and we need to seek some help, what would we do? So it follows a very similar trajectory as face‐to‐face, but there's just a couple of different considerations’. (Interview 10, both).

Telehealth was thus positioned as an alternative mode for delivering a very similar service as would be provided face‐to‐face. Indeed, many interviewees noted that their service did not have any specific policies regarding telehealth; rather, ‘it just tends to fall under our general privacy and confidentiality guidelines.’ (Interview 2, both). Consistent with this positioning, many interviewees noted that they gave clients the option to choose between face‐to‐face and telehealth appointments, either through their booking systems or verbally at the end of appointments:

‘Usually it's left to the client. I give them the option of phoning in or coming in’. (Interview 12, metropolitan).

However, some interviewees noted that this was at the discretion of the clinician, who could work with the client to decide on the most appropriate mode:

‘Sometimes it's up to the clinician to make a call on that. Like if there isn't good engagement, the clinician might recommend “Maybe it's better if we catch up face‐to‐face”’. (Interview 6, regional).

Consistent with the view that telehealth was simply an alternative mode of delivery, most interviewees reported that their service provided very little formal training for staff regarding conducting counselling via telehealth, beyond training in how to use telehealth platforms:

‘Initially when [video platform] was implemented, we had online training with a person that was from [video platform] … that was our main training, like how you use it, its different features’. (Interview 6, regional).

However, the absence of sensory cues and use of a less familiar platform could also mean that some staff lacked confidence in using telehealth. Interviewees described how their services used their clinical and operational supervision processes to support staff to develop skills in engaging and working therapeutically with clients at a distance:

‘No formal training, but a lot of support through operational and clinical supervision. Mostly around the barriers of telehealth such as keeping your client present, not being able to observe body language [so] being able to pick up on verbal cues’. (Interview 15, metropolitan).

Interviewees noted that, as with clients, clinician preferences for face‐to‐face or telehealth sessions varied. However, many interviewees felt that clinicians had a duty to offer telehealth services regardless of their personal preferences, because it was meeting a need for clients:

‘I used to avoid it, because I personally didn't like it … now I do see the value in it. I still don't like it, [but] from a client's perspective, it's reaching out to clients who wouldn't normally get that service’. (Interview 13, both).

## 4 Discussion

Overall, most interviewees viewed telehealth as an important (albeit not necessarily major) part of their service offering because it could increase access for clients who had difficulties reaching or did not feel comfortable attending face‐to‐face services. As such, telehealth was seen to offer a comparative advantage in addressing the high level of unmet need for AOD treatment services across Australia [2]. Standalone online counselling has been shown to attract a different demographic of clients in Australia: those who are female, younger, and more likely to access services outside of business hours [7]. Consistent with this study, telehealth and online counselling services have been found to reach people who are less likely to seek help in ‘traditional’ face‐to‐face services, due to concerns with stigma and shame, care or work responsibilities during business hours, geographical isolation, and long waiting lists [7, 35]. In a trial of a standalone telephone counselling service for alcohol use problems, 70% of participants had not previously accessed treatment, including those with complex barriers to treatment access comprising stigma, structural, attitudinal, and readiness‐to‐change barriers [36]. Telehealth may provide an important entry point into AOD treatment for some clients, with phone‐based services in particular viewed as providing additional psychological safety when commencing counselling. However, interviewees did not see telehealth as suitable for all clients, including clients whose privacy could not be ensured. In addition, some clients were precluded from accessing video‐based services due to limited resources. There is a need to explore alternative models for fostering digital inclusion while ensuring client privacy, for example by working with community services in regional areas to establish secure office spaces from which clients can access telehealth. It should be acknowledged that, at least for some service providers, telehealth was also seen as beneficial in increasing access because it overcame limitations in the frequency and availability of their face‐to‐face services. Other approaches to addressing unmet need, such as increasing face‐to‐face service provision in under‐served areas such as rural and remote regions and addressing stigmatised attitudes among health professionals [37] and in media reporting [38] also warrant continued focus.

Telehealth was viewed as relatively compatible with existing practices for face‐to‐face counselling, with challenges such as building a therapeutic alliance with clients [29] seen as something that could be addressed through general approaches to staff skill development, such as clinical and operational supervision—reflecting that client engagement is a common concern in AOD treatment settings more broadly [39]. However, interviewees' observations that counselling via telehealth uses different techniques to face‐to‐face suggests that telehealth‐specific training may also be warranted. There is a need for research to understand the therapist characteristics or skills required to effectively deliver telehealth, to support the development of evidence‐based training. In addition, there is a need for further research regarding the validity of risk assessments conducted over phone or video in an AOD setting. Concerns about risk assessments had prompted some services to encourage initial appointments to be held face‐to‐face, regardless of client preferences. A review of general diagnostic assessments via telehealth found that diagnostic accuracy via history‐taking or verbal assessment tools only (such as depression rating scales or neurocognitive tests) appears to be similar to face‐to‐face [12], but there has been limited AOD‐specific research. This research would aid service providers to develop policies and processes regarding assessment that appropriately balance client safety with access—including access during a crisis.

Many interviewees expressed uncertainty about the effectiveness of AOD counselling via telehealth compared to face‐to‐face. More research assessing outcomes is warranted but will need to take into account that the two modes may attract different cohorts of clients. Thus, randomised controlled trials comparing telehealth to face‐to‐face services will only provide part of the picture if they require participants to be willing and able to attend either a face‐to‐face or telehealth‐based service. Outcomes should be triangulated with trials involving control groups that do not require physical attendance (e.g., information, app‐based support) and real‐world outcomes data from service delivery. Outcomes may also differ between interventions that have been purposely designed and tested to be delivered through telehealth (e.g., [19]) and hybrid models of delivering standard care via a different modality, as commonly implemented by the services involved in this study.

### 4.1 Strengths and Limitations

This study provided insight into the real‐world implementation of telehealth services alongside face‐to‐face AOD counselling, outside of the constraints of clinical trials or pandemic restrictions. The key informant interview design allowed us to collect rich information regarding the experiences and perspectives of service providers. The sample included key informants from publicly funded specialist service providers located across Australia, including both metropolitan and regional locations, but was not intended to generalise to all service providers; government providers were under represented and the study did not include private providers. The sample included representatives from Aboriginal community controlled organisations, but was not designed to examine cultural nuances of telehealth delivery. Future studies involving Aboriginal research leadership and governance and employing culturally appropriate research methods are required. It will also be important for future studies to triangulate service provider perspectives with those of clients, as well as quantitative measures of the demographic profile and outcomes of clients who use telehealth.

## 5 Conclusion

Offering telehealth alongside face‐to‐face counselling services may offer a comparative advantage over delivering face‐to‐face treatment alone, by making it physically and psychologically easier for some clients to access services. Service providers viewed telehealth as relatively compatible with current processes and something that could be implemented flexibly depending on client preferences. However, uncertainties around comparative clinical effectiveness and safety, particularly via phone, led some services to proceed with caution. Further research into therapist skills, tools, and models of care is required to support the development of evidence‐based training and guidance for AOD services in how to deliver safe and effective counselling via telehealth.

## Author Contributions

Each author certifies that their contribution to this work meets the standards of the International Committee of Medical Journal Editors.

## Conflicts of Interest

The authors declare no conflicts of interest.

## Data Availability

Research data are not shared due to privacy and ethical considerations.
